# Modeling and Optimization of Sensitivity and Creep for Multi-Component Sensing Materials

**DOI:** 10.3390/nano13020298

**Published:** 2023-01-11

**Authors:** Gangping Bi, Bowen Xiao, Yuanchang Lin, Shaoqiu Yan, Ying Tang, Songxiying He, Mingsheng Shang, Guotian He

**Affiliations:** 1 Chongqing Institute of Green Intelligent Technology, Chinese Academy of Sciences, Chongqing 400714, China; 2 College of Mechanical Engineering, Chongqing University of Technology, Chongqing 400054, China; 3 Chongqing Key Laboratory of Artificial Intelligence and Service Robot Control Technology, Chongqing Institute of Green Intelligent Technology, Chinese Academy of Sciences, Chongqing 400714, China; 4 Chongqing School, College of Artificial Intelligence, University of Chinese Academy of Sciences, Chongqing 400020, China

**Keywords:** RSM, SVR, NSGA-II, MWCNTs, MLG, Ni, magnetic field, sensitivity, creep

## Abstract

Pressure sensors urgently need high-performance sensing materials in order to be developed further. Sensitivity and creep are regarded as two key indices for assessing a sensor’s performance. For the design and optimization of sensing materials, an accurate estimation of the impact of several parameters on sensitivity and creep is essential. In this study, sensitivity and creep were predicted using the response surface methodology (RSM) and support vector regression (SVR), respectively. The input parameters were the concentrations of nickel (Ni) particles, multiwalled carbon nanotubes (MWCNTs), and multilayer graphene (MLG), as well as the magnetic field intensity (B). According to statistical measures, the SVR model exhibited a greater level of predictability and accuracy. The non-dominated sorting genetic-II algorithm (NSGA-II) was used to generate the Pareto-optimal fronts, and decision-making was used to determine the final optimal solution. With these conditions, the optimized results revealed an improved performance compared to the earlier study, with an average sensitivity of 0.059 kPa^−1^ in the pressure range of 0–16 kPa and a creep of 0.0325, which showed better sensitivity in a wider range compared to previous work. The theoretical sensitivity and creep were relatively similar to the actual values, with relative deviations of 0.317% and 0.307% after simulation and experimental verification. Future research for transducer performance optimization can make use of the provided methodology because it is representative.

## 1. Introduction

Due to their potential applications in a variety of industries, including the aviation, space, chemical, automotive, and biomedicine industries [1,2,3,4], polymer nanocomposites have become a hot topic of study. Conducting polymer nanocomposites are used in numerous sensor applications to achieve intelligence, informationalization, and the future networks [5]. Performance characteristics of sensors include sensitivity, creep, repeatability, hysteresis [6], etc. Sensitivity and creep have become the two key metrics affecting the performance of sensors, since they influence measurement accuracy and stability, respectively. However, the fabrication of materials for outstanding sensitivity and creep resistance is heavily constrained by the complexity of the sensing system.

A promising method for enhancing the sensitivity and creep resistance has been identified as properly adjusting the choice of materials and the reasonable ratio. For a piezo-resistive tactile sensor, conductive filler particles are typically chosen from materials with the same dimensions, such as Ni (zero dimensions), CNT (one dimension), MLG (two dimensions), and others. High sensitivity and creep resistance for sensors have been the focus of an intense research effort. Xu et al. [7] produced CNT/PDMS composites to obtain great sensitivity in terms of single particle filling. The interface connection and progressive deterioration of CNT-based polymer composites were shown by Xia X et al. [8] to be major influences on the creep, and CNT could also be employed to increase the creep resistance of composites. Sun R. et al. [9] investigated the impact of MWCNTs and Ni on nanocomposites with regard to two-particle filling. To attain high sensitivity, a synergistic effect between two particles occurred during the experiment studying the rearrangement reaction between Ni and MWCNTs under a magnetic field. The impact of MLGs, CNTs and their combination on sensitivity was investigated by Ali A. et al. [10]. Their findings demonstrated that composites with mixed fillings have better sensitivity than composites with single-particle filling. The impact of CNTs, MLGs, and their combination on creep resistance was investigated by Charito et al. [11]. The outcome showed that the creep resistance can be improved by mixing CNTs and MLGs. The impact of a magnetic field on MWCNT/Ni composites blended in various ratios was investigated by Lu C. et al. [12]. The outcome of their study demonstrated that a stronger magnetic field can improve homogeneity, which further raises a composites’ conductivity. Chen Y. et al. [13] developed a sensor based on CNT/Ni/PMXene composites for three-particle filling. They demonstrated how three materials interacting create a one- and two-dimensional integrated conductive network, greatly enhancing the sensitivity of composites. Table 1 displays past efforts to enhance a sensor’s performance. It can be shown that the majority of the extant literature, particularly for single-particle filling and two-particle filling, has extensively evaluated the influence of filling particles on sensitivity or creep alone. There is not much research that has examined how particle selection affects creep and sensitivity. According to the available research, the combination of filler particles with various shapes and characteristics does improve sensitivity and creep resistance. The combination of Ni and CNT can increase the sensitivity of sensors [9,12,13], the mixture of CNT and MLG can increase sensitivity and creep resistance [10,11], and the magnetic field can somewhat improve the performance of composites by increasing uniformity [9,12]. The combination of the aforementioned materials is, in theory, favorable to both sensitivity and creep. To our knowledge, no performance analysis of a combination of Ni, CNT, and MLG has been published. Therefore, it is worthwhile to conduct research on both sensitivities and creep using the above materials in combination.

The additive proportion of these materials is still difficult to determine because of the complexity of the synergistic effect and reaction mechanism among filling particles. Due to several factors, it is a time-consuming and expensive operation to estimate the manufacturing conditions without a mathematical model. In the area of material synthesis and process optimization, analysis and optimization of traditional experimental synthesis methods have been significant subjects of research. One of the most used statistical analysis models, RSM, was developed by Box and Wilson in 1951 [14], and is used to evaluate and forecast many experimental conditions. RSM, which is widely used in the food and chemical fields [15], creates prediction models to assess the single factor or interaction of each factor based on a small number of trials. However, due to the complex nonlinear situation, the RSM quadratic polynomial occasionally fails to provide the required forecast accuracy. In comparison to RSM, the SVR model has a good prediction of non-linear and unconnected data as a nonlinear regression model, which improves the speed of operation by transforming the objective function, improving the conditions of the equations, and lowering the complexity of the computation [16]. It has been widely used in many engineering fields. Considering the high accuracy of SVR, it may overcome the disadvantages of RSM. Although RSM and SVR models have been utilized for process prediction in the food and chemical fields, few studies have used RSM or SVR models in the sensor field to estimate and analyze performance. Thus, such a technique deserves study.

This study represents the first attempt to model and optimize the sensitivity and creep resistance of multi-component conductive nanocomposites with a magnetic field, i.e., Ni, MWCNT, and MLG, under experimental conditions. RSM and SVR algorithms were used to forecast the experimental input conditions and the output performances, respectively. The NSGA-II method was used to determine the Pareto-optimal fronts for the experimental settings, and decision-making was used to choose the best option. This work illustrated the applicability of the suggested approach for performance prediction and optimization in the realm of intelligent sensing.

## 2. Theory and Methods

### 2.1. Samples Preparation, Fabrication, Data Acquisition, and Interior Structure

#### 2.1.1. Preparation of Materials

The matrix was synthesized from dimethyl silicone oil, room-temperature vulcanized silicone rubber (RTV), and a curing agent purchased from Hong Ye-Jie Technology, China. Ni particles, with an average diameter of 500 nm and purity of 99.9 wt%, were supplied by Hang-Ba Metal Materials Co., Ltd., China, and used as the soft magnetic materials along with the conductive phase. MWCNTs were obtained from Suzhou Tan-Feng Graphene Technology Co., Ltd., China. The purity of the MWCNTs was larger than 95 wt%, their length was larger than 3 um, and their outer diameter was 8–15 nm. MLGs with 5–10 layers and an average diameter of 50 um were provided by Suzhou Tan-Feng Graphene Technology Co., Ltd., Suzhou, China.

#### 2.1.2. Fabrication of Materials

**Figure 1:**
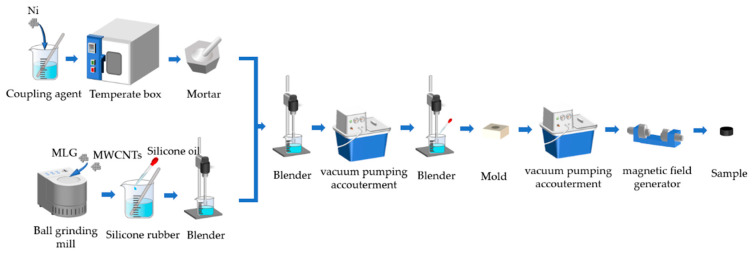
The fabrication process of the materials.

Figure 1 illustrates the fabrication processes of the composites. Before the fabrication process began, three materials were successively weighed on an electronic balance. After that, Ni nanoparticle surfaces were pretreated by a coupling agent. Then, Ni was heated at a constant temperature of 80 °C for 12 h and subsequently ground into a powder. At the same time, MWCNTs and MLGs were ground in a ball-grinding mill to achieve uniform separation. Then, RTV and dimethyl silicone oil were proportionally mixed with ground MWCNTs and MLGs and added into a beaker to be evenly stirred for 10 min with a mechanical mixer at 2300 rpm for filler dispersion. After that, the ground Ni was added into a beaker and evenly stirred for 10 min for uniform distribution. After 10 min of mixing, the interior of the mixture was full of air bubbles that were eliminated by vacuum pumping. Afterwards, the curing agent was added to the mixture in a specific proportion and stirred for uniform mixing. To form the desired shape, the mixture was poured into a mold, and meanwhile the remaining air bubbles has been released. The pumping time should be short enough to avoid solidification before extrusion forming. Finally, the mold was fixed in a magnetic field generator under a magnetic field. The desired samples were obtained after they were fully cured.

#### 2.1.3. Acquisition of Raw Data

The composites’ sensitivity and creep were assessed under various conditions. The testing apparatus included a computer, an LCR meter, the created samples, and a press. Samples were placed between two metal plates for testing. Under uniaxial pressing, piezoresistive measurements were made. For simultaneous stress-resistance measurements, a constant compression speed was maintained. To analyze the sensitivity performance, the actual pressures that the samples encountered were recorded and the resistances under those pressures were computed. The pressure was constant during the entire process and the resistances for the corresponding time were recorded to test creep performance. The methodologies for creep measurements were comparable to those for the piezoresistive tests.

#### 2.1.4. Interior Structure of Composite

**Figure 2:**
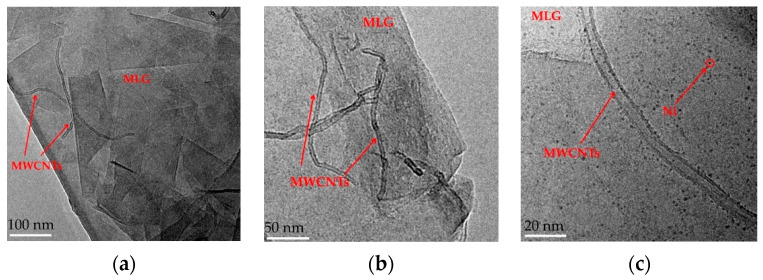
TEM images of composites. (**a**) TEM plot of Ni/CNT/MLG particles at 100 nm scale. **(b**) TEM plot of Ni/CNT/MLG particles at 50 nm scale. (**c**) TEM plot of Ni/CNT/MLG particles at 20 nm scale.

Figure 2a shows the TEM images of the fabricated composite with different magnifications. As shown in Figure 2a,b, the MLGs were stacked layer by layer and the MWCNTs were distributed evenly between two layers. Figure 2c shows a more detailed view; illustrating that the Ni particles were adsorbed on the surface of CNTs and MLGs at the same time.

### 2.2. General Mechanisms of Sensitivity and Creep

#### 2.2.1. Sensitivity Mechanism

The sensitivity is linked to many electronic conduction theories, such as the field emission theory, tunneling effect theory, and conduction path theory [15]. In the conductive path theory, the current channel is produced through contact between neighboring conductive particles. The tunneling effect theory states that an electronic transition carried out by thermal agitation results in a current passing via brief gaps between nearby particles. According to the field emission hypothesis, an external electric field creates an electric field among conductive particles and the electrons then overcome the potential barrier to create a current. A high voltage is necessary during the entire procedure. The tunneling effect theory and the impact of electric conduction mechanisms are the main theories considered in this study.

**Figure 3:**
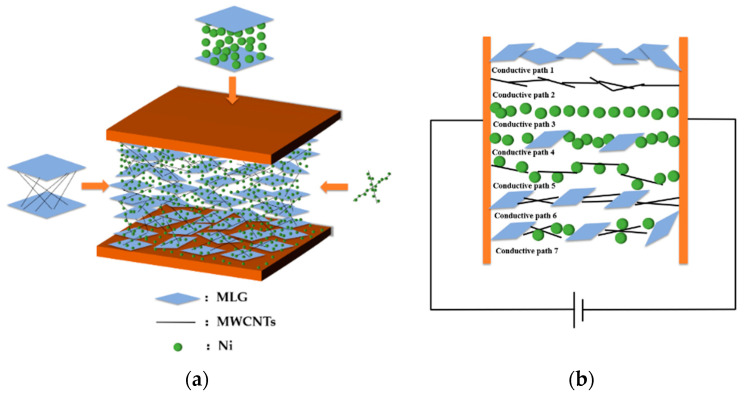
The microstructure of composites and conductive pathways. (**a**) The theoretical microstructure model of Ni/CNT/MLG composites and (**b**) the conductive paths during compression.

According to Figure 3, MWCNTs are disseminated in the matrix between neighboring MLG nanoplates when combined with MLG to improve conductivity. In particular, tangled MWCNTs fill the spaces between nearby MLG plates, which promotes the development of current channels. Similar to the preceding process, the combination of MLG and Ni causes Ni nanoparticles to scatter in the spaces between MLG plates, accelerating the development of a conductive network. When MWCNTs and Ni particles are combined, the particles form a structure resembling a “bunch of grapes” on the surface of the CNTs. This connection between the CNTs improves the conductivity of the composites. In this research, the above three materials achieved a novel conductivity. The theoretical model is shown in Figure 3a.

**Figure 4:**
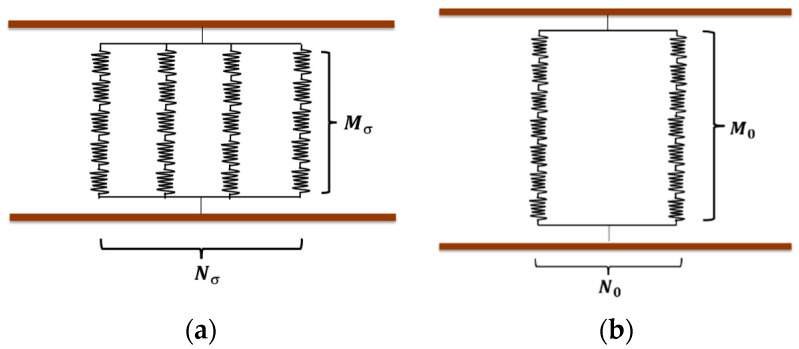
Piezoresistive effect for composites. (**a**) Equivalent circuit without compression and (**b**) equivalent circuit with compression.

Such a theoretical model was developed based on earlier research. Before curing, the magnetic field causes materials to be equally distributed in the same direction throughout the matrix. In particular, MLGs disperse uniformly throughout the matrix, MWCNTs are dispersed in the spaces between MLG nanoplates, and Ni nanoparticles are dispersed throughout the matrix, some of which are dispersed in the spaces between MLG plates and some of which are adsorbed on the surface of CNTs and MLGs. These distributions interact to create the current paths depicted in Figure 3b. Due to the numerous conductive paths created by such a complex distribution, the resistance produced is correlated with the material’s characteristics, the distance and conductive area between particles, the number of particles in a single conductive pathway, and the number of conductive pathways. The analogous circuit of the piezoresistive effect for composites is depicted in Figure 4. A decrease in the number of particles per conductive channel and an increase in the total number of conductive pathways are caused by an increase in pressure, and this results in a drop in resistance.

According to the tunneling effect theory, the electrical resistance of the conductive composites can be described as [17,18]:

(1) $$R=\frac{M}{N}\frac{8\pi hd}{3S\gamma e^{2}}\exp\left(\gamma d\right)$$

(2) $$\gamma =\frac{4\pi }{h}\sqrt{2m\varphi }$$

where R is composite resistance, M is the number of particles on one single current pathway, N is the number of effective conductive pathways, h is Plank’s constant, e is the electron charge, d is the minimum distance between two particles, S is the effective cross-sectional area between neighboring particles, *m* is the electron mass, and φ is the height of the potential barrier.

During the compression process, suppose the minimum distance decreases from $d_{0}$ to d under uniaxial compression, where d is based on the deformation of the matrix and the deformation of particles is ignored, so:

(3) $$\varepsilon =\frac{d_{0}-d}{d_{0}}$$

where *ε* represents the composite strain.

Substituting Equations (2) and (3) into (1), Equation (4) shows the relationship between the original and changed resistance:

(4) $$\frac{R}{R_{0}}=\frac{M\left(\sigma \right)N\left(0\right)}{M\left(0\right)N\left(\sigma \right)}\left(1-\varepsilon \right)\exp\left(-\gamma d_{0}\varepsilon \right)$$

where R denotes the resistance under compression, $R_{0}$ denotes the resistance without compression, and $\frac{M\left(\sigma \right)N\left(0\right)}{M\left(0\right)N\left(\sigma \right)}$ represents the degree of particle rearrangement.

Thus, the expression for the relative change in resistance can be derived:

(5) $$\frac{\Delta R}{R_{0}}=1-\frac{M\left(\sigma \right)N\left(0\right)}{M\left(0\right)N\left(\sigma \right)}\left(1-\frac{\sigma }{E}\right)\exp\left(-\gamma d_{0}\frac{\sigma }{E}\right)$$

where *E* denotes Young’s modulus, and ΔR denotes ($R_{0}-R$).

Since the strain and stress of composites are nonlinearly related, the elastic modulus changes under compression. To increase model accuracy, changes in Young’s modulus are considered, which is expressed through the following polynomial function:

(6) $$E=E_{0}+B_{1}\sigma +B_{2}\sigma^{2}$$

where $E_{0}$ denotes the original Young’s modulus, and $B_{1}$ and $B_{2}$ are constants.

Substituting Equation (6) into (5), the expression related to sensitivity is shown, where the sensitivity is the ratio between the relative change in resistance, $\frac{\Delta R}{R_{0}}$, and pressure, σ:

(7) $$\frac{\Delta R}{R_{0}}=1-\frac{M\left(\sigma \right)N\left(0\right)}{M\left(0\right)N\left(\sigma \right)}\times \left(1-\frac{\sigma }{E_{0}+B_{1}\sigma +B_{2}\sigma^{2}}\right)\exp\left(-\frac{\gamma d_{0}\sigma }{E_{0}+B_{1}\sigma +B_{2}\sigma^{2}}\right)$$

From Equation (7), the sensitivity is associated with the degree of particle rearrangement, Young’s modulus of materials, and the distance between each filler, which is affected by the nature and contents of fillers and the fabrication process. Thus, in this study, the contents of Ni, MWCNTs, and MLGs and the magnetic field intensity are assumed as the four experimental conditions that influence the sensitivity of the composites. A discussion of the influences of each factor on sensitivity is given in the following part.

#### 2.2.2. Creep Mechanism

The process known as creep occurs when the strain on a solid material grows over time while the stress remains constant. Instantaneous deformation, primary creep, and secondary creep are three stages that can be used to categorize the processes causing this occurrence. In this investigation, creep has a considerable impact on the resistance of the composites. As a result, the idea of “resistance creep” was proposed, which suggests that under sustained pressure, a material’s resistance changes with time. Even though the dependent variable is different, these two creep formulations are consistent with the same law. Therefore, to conduct further investigations, a transformation between strain and change in resistance is needed.

**Figure 5:**
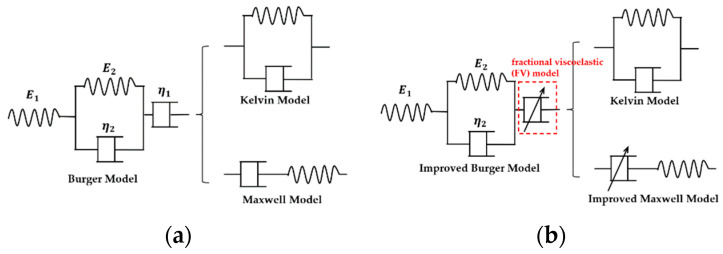
The expression model for creep. (**a**) The traditional Burger model; (**b**) the improved Burger model that integrates the FV model.

The Burger model, which is depicted in Figure 5a, is frequently used to describe the creep of solid materials and consists of the Maxwell model with the spring-damping model in series and the Kelvin model with the spring-damping model in parallel. In particular, the instantaneous deformation is represented by a spring in the Maxwell model, the main creep stage is expressed by the Kelvin model, and the secondary creep stage is shown by damping in the Maxwell model.

Equation (8) shows an expression for the Burger model [19]:

(8) $$\varepsilon \left(t\right)=\frac{\sigma }{E_{1}}+\frac{\sigma }{E_{2}}\left[1-\exp\left(-\frac{E_{2}}{\eta_{2}}t\right)\right]+\frac{\sigma }{\eta_{1}}t$$

where $E_{1}$ is Young’s modulus of spring in the Maxwell model, $E_{2}$ and $\eta_{2}$ indicate Young’s modulus and viscosity in the Kelvin model, respectively, and $\eta_{1}$ represents the viscosity of damping in the Maxwell model.

However, in an actual experiment, complete viscosity is impossible, which means the elasticity still affects the creep of materials in the last stage. Since damping in the Maxwell model is not suitable to predict the secondary creep process, the fractional viscoelastic (FV) model is used for predicting the last stage. The improved model is shown in Figure 5b.

The stress–strain relationship of the elastic and viscous elements are defined as [20]:

(9) $$\sigma \left(t\right)=E\tau^{\alpha }\frac{d^{\alpha }}{dt^{\alpha }}\varepsilon \left(t\right)\;or\;\varepsilon \left(t\right)=\frac{1}{E\tau^{\alpha }}\frac{d^{-\alpha }}{dt^{-\alpha }}\sigma \left(t\right)\;0\leq \alpha \leq 1$$

where *α* is the order of derivative; when *α* = 0, the fractional viscous element degenerates into an elastic element; when *α* = 1, it becomes a viscous element. $\varepsilon \left(t\right)$ and $\sigma \left(t\right)$ are the strain and stress at a given time *t*, $\frac{d^{\alpha }}{dt^{\alpha }}$ is the differentiation of the order α at time t, τ=η/E is the relaxation time, η is the viscosity, and E is the stiffness of the material.

Following this equation shows a property of the Fractional order model [21]:

(10) $$\frac{d^{k}t^{j}}{dt^{k}}=\frac{\Gamma \left(j+1\right)}{\Gamma \left(j-k+1\right)}t^{j-k}$$

where k is positive for differentiation and negative for integration and Γ represents the gamma function.

In this study, strain was calculated through the integration of constant stress to order *α*. Thus, in Equation (10), j=0, k=−α, and $\Gamma \left(1\right)=1$, and the strain response becomes:

(11) $$\varepsilon =\frac{\sigma_{0}}{E}\frac{1}{\Gamma \left(1+\alpha \right)}{\left(\frac{t}{\tau }\right)}^{\alpha }$$

where $\sigma_{0}$ is the constant stress.

Substituting Equation (11) into Equation (8), the expression for improved Burger model is:

(12) $$\varepsilon \left(t\right)=\frac{\sigma }{E_{1}}+\frac{\sigma }{E_{2}}\left[1-exp\left(-\frac{E_{2}}{\eta_{2}}t\right)\right]+\frac{\sigma_{0}}{E}\frac{1}{\Gamma \left(1+\alpha \right)}{\left(\frac{t}{\tau }\right)}^{\alpha }$$

To analyze the relationship between strain and change in resistance, the change in the number of effective conductive pathways under pressure is given as [22]:

(13) $$N\left(\sigma \right)=N\left(0\right)\exp\left(C_{1}\varepsilon +C_{2}\varepsilon^{2}+C_{3}\varepsilon^{3}\right)$$

where $N\left(\sigma \right)$ and $N\left(0\right)$ are the number of effective conductive pathways after and before compression, respectively.

Since *d*_0_ is extremely small, resulting in a value of $\gamma d_{0}≅0$, from Equations (1)–(3) and (13), the relationship between $\frac{\Delta R}{R_{0}}$ and ε is:

(14) $$\frac{\Delta R}{R_{0}}=1-\frac{\left(1-\varepsilon \right)}{\exp\left(C_{1}\varepsilon +C_{2}\varepsilon^{2}+C_{3}\varepsilon^{3}\right)}$$

where $C_{1}$, $C_{2}$_,_ and $C_{3}$ are adjustable parameters.

Substituting Equation (12) into Equation (14), the expression for resistance creep is proposed as:

(15) $$\frac{\Delta R}{R_{0}}=1-\frac{\left(1-\varepsilon \left(t\right)\right)}{\exp\left(C_{1}\varepsilon \left(t\right)+C_{2}\varepsilon {\left(t\right)}^{2}+C_{3}\varepsilon {\left(t\right)}^{3}\right)}$$

where $\varepsilon \left(t\right)$ is the creep based on the improved Burger model.

According to Equation (15), the Young’s modulus and viscosity of materials are related to creep, which is also influenced by the types and composition of materials as well as the manufacturing process. This is because different materials with different compositions can be combined to produce different properties. As a result, the following procedure considers the Ni content, MWCNTs, MLG, and magnetic field intensity as the four experimental factors that can affect how quickly composites creep. In the section that follows, each factor’s effects on creep are discussed and studied.

### 2.3. Prediction and Optimization Approaches

#### 2.3.1. Response Surface Methodology (RSM)

**Figure 6:**
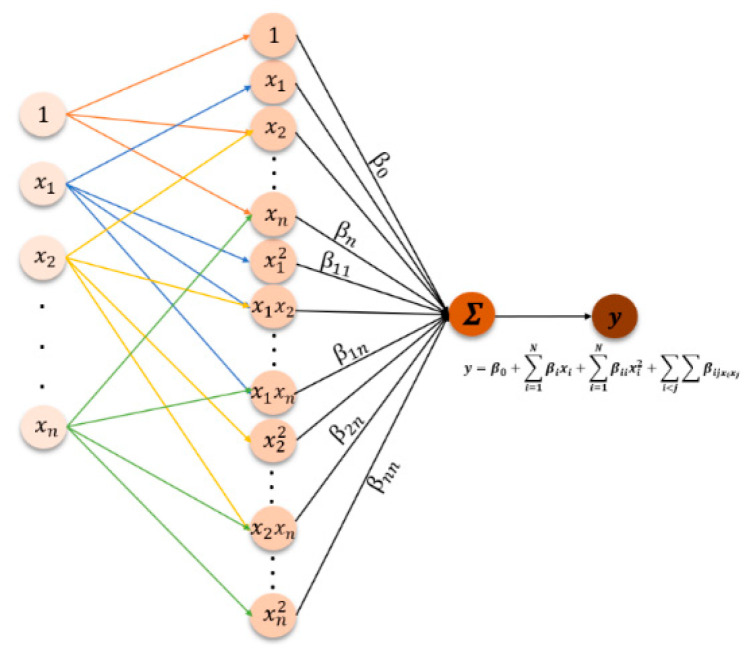
Schematic view of the RSM model.

To evaluate the relationship between one or more outputs and the input parameters, the response surface method (RSM), a modeling-based relatively basic tool with low computational burden [23], uses a randomized and unbiased design. Equation (16) presents the general mathematical relationship between input parameters and output variables in RSM, and Figure 6 shows a detailed schematic view:

(16) $$y=\beta_{0}+\overset{N}{\underset{i=1}{\sum }}\beta_{i}x_{i}+\overset{N}{\underset{i=1}{\sum }}\beta_{ii}x_{i}^{2}+\underset{i<j}{\sum }\sum \beta_{ijx_{i}x_{j}}$$

where *y* is the response variable, *x_i_* is the input parameters, $\beta_{0}$ denotes the bias, $\beta_{i}$ is the linear effect, $\beta_{ii}$ represents the squared effect, and $\beta_{ij}$ is the interaction effect.

RSM is divided into two categories: the central composite design (CCD) and the Box–Behnken design (BBD), both of which are popular and useful in laboratory trials. The BBD technique was selected for this research to effectively acquire optimal conditions. BBD can be thought of as three interlocking factorial designs with three coded levels for each input parameter, such as −1, 0, and +1, provided by the center points.

To forecast and optimize the sensitivity and creep in this work, four significant factors—Ni content, MWCNT content, MLG content, and magnetic field intensity—were chosen as the input parameters. Specifically, single-factor trials yielded the range (detailed data are illustrated in Figures S1–S4). Table 2 displays the specific level of each input. Table 3 displays the experimental findings under various fabrication settings. The resistance creep stabilizes for all situations within 120 s in Figures S1–S4. For all situations, the relative change in resistance rises from 0 to 16 kPa. As a result, for the sake of the analysis that follows, the measured range for creep is taken to be 120 s, and the pressure range of sensitivity is taken to be 0–16 kPa. To be more precise, as this change represents a nonlinear variation in the real experiment within the range of 0 to 16 kPa, the relative change in resistance under 16 kPa is taken as the average sensitivity. Resistance creep was defined as the relative variation in resistance under continuous pressure from 0 to 120 s (hereafter referred to as creep). The ratio of the relative change in resistance and pressure is used to calculate the actual sensitivity, which is the goal measurement. The relative change in resistance is directly taken into account as the sensitivity evaluation index because the pressure for each condition is 16 kPa (hereafter referred to as sensitivity).

#### 2.3.2. Support Vector Regression (SVR)

**Figure 7:**
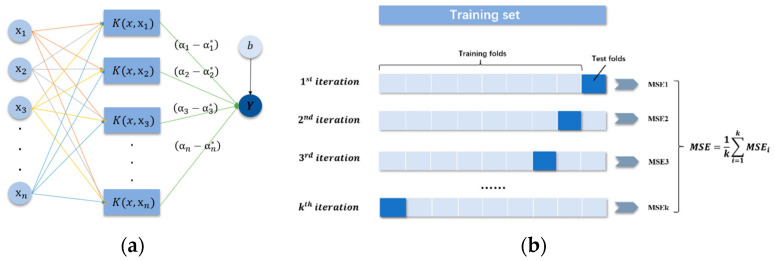
(**a**) Schematic view of the SVR model and (**b**) k-fold cross-validation approach.

The SVR is a potent artificial intelligence technique that calculates the relationship between inputs and outputs through a suitable hyperplane with fewer error bounds and may be utilized as a powerful modeling tool for predicting process performance in a variety of engineering fields. Equation (17) depicts the SVR model’s general equation, and Figure 7a shows the model’s schematic perspective, the specific derivation process are based on Equations (A4)–(A9) (Appendix A).

(17) $$y=\overset{n}{\underset{i=1}{\sum }}\left(\alpha_{i}-\alpha_{i}^{*}\right)K\left(x,x_{i}\right)+b$$

where ($\alpha_{i}$, $\alpha_{i}^{*}$) represents the Lagrange multipliers utilized for dual space transformation and $K\left(x,x_{i}\right)$ depicts the kernel function used to solve quadratic equations. The kernel functions are used to solve a linearly inseparable problem which divides into three parts: linear, polynomial, and gaussian, and *b* depicts the bias.

Additionally, the hyperparameters also play a significant role in the performance of the SVR model, such as gamma, C, and epsilon. Hence, the design of hyperparameters is an exceedingly key part of SVR modeling. In this research, the gaussian kernel function was selected, the grid search algorithm was carried out to tune the hyperparameters, and a five-fold cross-validation approach was utilized for data partitioning, as shown in Figure 7b.

### 2.4. NSGA-II Optimization Approach

**Figure 8:**
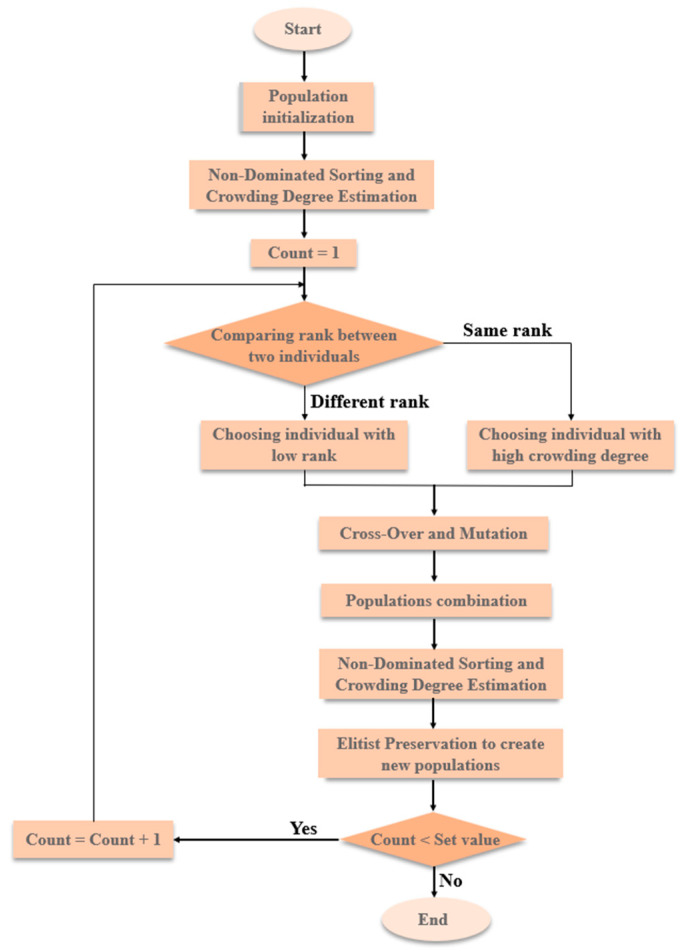
The algorithm flow for NSGA-II.

NSGA-II determines the Pareto optimal set of multi-objective and multi-constraint problems by calculating the congestion distance of parameters [24], which introduces the concept of the individual crowding degree and combines the tournament method along with the elite retention strategy to act on the selection operator to choose the individuals entering the next generation population [25]. The specific algorithm is shown in Figure 8. In this study, the sensitivity and creep were considered as two optimized objects. Through such a method, the Pareto optimal set for two performances can be solved.

## 3. Results and Discussion

### 3.1. Models Establishment by RSM

#### 3.1.1. Experimental Results Analysis for Sensitivity

The experimental data were analyzed using Design-Expert software. To evaluate the accuracy of the fitting and determine the strength of the influence of the input parameters on the output variables, the analysis of variance (ANOVA) was developed. Table S1 displays the sensitivity ANOVA findings (Supplemental Materials). As can be seen, the regression model’s significant value (*p*-value) is less than 0.0001 and the lack of fit item’s *p*-value is more than 0.05, indicating that the regression model is highly significant and the lack of fit item is not significant. The RSM model’s R^2^ score of 0.9865 demonstrated the model’s high level of fit. This regression model’s coefficient of variance (C.V.%) was 2.91%, lower than 10%, indicating the agreement of results. Additionally, each element’s *p*-value was less than 0.0001, demonstrating the high influence each factor has on sensitivity. The order of significance based on the F-value is MWCNT > MLG > Ni > B. Following the BBD’s ANOVA regression coefficients, the following can be used to derive the link between the sensitivity and the input parameters:

$$\begin{array}{ll} y_{1}=0.662967 & -0.234823x_{1}+1.45172x_{2}-0.875818x_{3}+0.217583x_{4}-0.621684x_{1}x_{2}\\ & +0.644648x_{1}x_{3}+0.108931x_{1}x_{4}+1.01565x_{2}x_{3}+0.093508x_{2}x_{4}\\ & -0.363485x_{3}x_{4}-0.026424x_{1}^{2}0.743547x_{2}^{2}-0.275761x_{3}^{2}-0.030284x_{4}^{2} \end{array}$$

where $y_{1}$ indicates the sensitivity, $x_{1}$ indicates the content of Ni, $x_{2}$ represents the content of MWCNT, $x_{3}$ represents the content of MLG, and $x_{4}$ is the magnetic field intensity, B.

**Figure 9:**
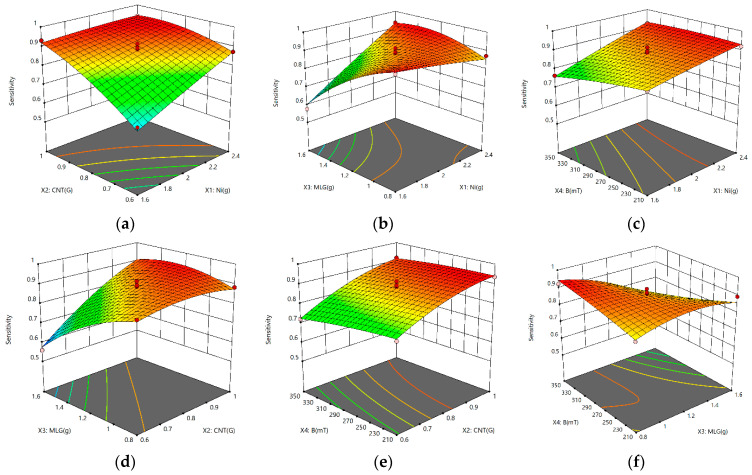
3D response surface plots. (**a**) Interaction between the MWCNT and Ni content; (**b**) interaction between the MLG and Ni content; (**c**) interaction between magnetic field intensity (B) and Ni content; (**d**) interaction between MLG and MWCNT content; (**e**) interaction between MWCNT content and B; (**f**) interaction between MLG content and B.

The interactions between the factors on the sensitivity are illustrated in Figure 9. When the interaction between two factors was studied, the other two factors were at center levels.

In Figure 9a–f, the 3D response surface plots are displayed. Figure 9b,d,f show how the interactions between MLGs and Ni, CNTs, and B have a clear effect on sensitivity. The relationship between Ni and CNTs, MLG, and B are depicted in Figure 9a–c. Increased sensitivity results from an increase in Ni content. The sensitivity of composites peaked at 2.4 g of Ni, demonstrating that Ni, when mixed with CNTs and MLG under specific magnetic fields, offers increased conductivity. Figure 9a,d,e show how CNTs interact with Ni, MLG, and B, showing that the sensitivity rises as CNT content increases. When the CNT content reaches 1 g, the sensitivity reaches a maximum. To increase the synergistic effect when interacting with MLG and Ni, CNTs can be used. According to Figure 9b,d,f, as the MLG content is increased, the sensitivity first rises before falling. The interplay of these elements had a combined influence on the sensitivity based on the aforementioned results and study; hence, a suitable percentage of these factors must be present to obtain high sensitivity.

#### 3.1.2. Experimental Results Analysis for Creep

Table S3 displays the ANOVA findings for creep (Supplemental Materials). The regression model’s significant value (*p*-value) is less than 0.0001 and the lack of fit item’s *p*-value is greater than 0.05, which shows that the model is reasonable and useful. The RSM model’s R^2^ score was 0.9774, indicating that the model was well-fitted. The regression model’s coefficient of variance (C.V.%) was 7.898%, less than 10%, indicating that the outcomes were consistent. Additionally, the *p*-values for CNT, MLG, Ni, and B are all less than 0.0001 and less than 0.05, demonstrating the high impact of each element on creep. The order of significance based on the F-value is MWCNT > MLG > B > Ni. The relationship between the creep and the input parameters may be deduced from the BBD’s ANOVA regression coefficients as:

$$\begin{array}{ll} y_{2}= & 0.181745+0.122802x_{1}+0.006281x_{2}-0.171199x_{3}-0.187587x_{4}-0.022432x_{1}\\ & -0.026087x_{1}x_{3}+0.003694x_{1}x_{4}+0.02414x_{2}x_{3}+0.020221x_{2}x_{4}+0.017287x_{3}x_{4}\\ & -0.018577x_{1}^{2}+0.016595x_{2}^{2}+0.061551+0.037561x_{4}^{2} \end{array}$$

where $y_{2}$ indicates the creep, $x_{1}$ indicates the content of Ni, $x_{2}$ represents the content of MWCNT, $x_{3}$ represents the content of MLG, and $x_{4}$ is the magnetic field intensity, B.

**Figure 10:**
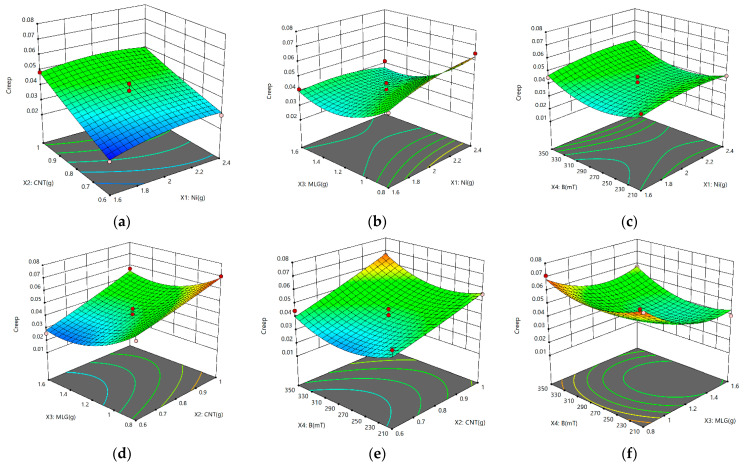
3D response surface plots. (**a**) Interaction between the MWCNT and Ni content; (**b**) interaction between the MLG and Ni content; (**c**) interaction between B and Ni content; (**d**) interaction between MLG and MWCNT content; (**e**) interaction between MWCNT content and B; (**f**) interaction between MLG content and B.

The interactions between the factors are illustrated in Figure 10. When the interaction between two factors was studied, the other two factors were at center levels.

Figure 10a,d,e display the findings of the interactions between CNTs and Ni, MLGs, and B, demonstrating that an increase in CNT content caused an increase in creep. Figure 10b,d,f show that when MLG interacts with other parameters, the creep first lowers and then grows as the MLG content rose. According to the preceding graphs, when additional elements are present, B and Ni contents have a small impact on creep. The findings and analysis show that there is a contradiction between the effects of contents on sensitivity and creep, which indicates that a persistent pursuit of high sensitivity may result in subpar creep performance. Therefore, there must be a trade-off between sensitivity and creep.

### 3.2. Model Establishment by the SVR Method

**Figure 11:**
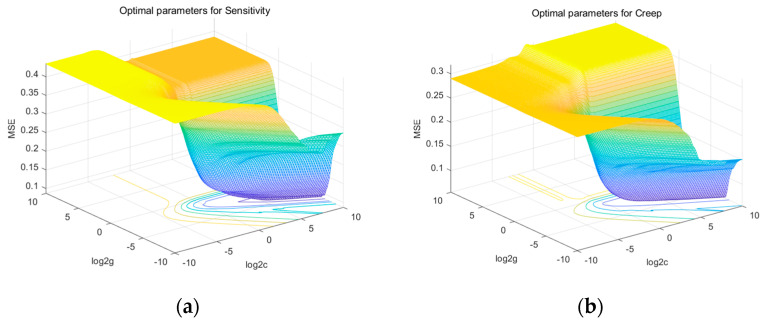
Optimal parameters results. (**a**) Parameter selection for sensitivity and (**b**) parameter selection for creep.

Ni, MWCNT, and MLG contents and B were used as input parameters in data samples based on RSM; sensitivity and creep were taken into consideration as forecastable output variables. The value of the average MSE obtained from a five-fold cross-validation of the sensitivity is the smallest when C was 59.7141 and gamma was 0.0508; for the creep, the smallest average MSE was determined using the grid search algorithm and the five-fold cross-validation method when C was 17.1487 and gamma was 0.0947. Figure 11 depicts the procedure, while Table 4 and Table 5 provide the R^2^, RMSE, and MAE values for the SVR model.

The average R^2^, RMSE, and MAE of 0.982, 0.031, and 0.02339, respectively, shown in Table 4 for the sensitivity evaluation indicated a strong connection between theoretical and experimental values, which are calculated based on Equations (A1)–(A3). Additionally, the R^2^, RMSE, and MAE ranges are 0.9726–0.9882, 0.0175–0.0403, and 0.01467–0.03205, respectively. These small ranges demonstrate the strong robustness of the SVR model. The average R^2^, RMSE, and MAE for the creep evaluation were 0.9656, 0.006139, and 0.005049, respectively, demonstrating the SVR model’s strong fitness. High resilience is also shown by the R^2^, RMSE, and MAE ranges, which are 0.9532–0.9789, 0.00356–0.00781, and 0.00258–0.0071, respectively. All the results show that the established SVR model effectively predicts the sensitivity and creep performances with high robustness. 

### 3.3. Comparison between Applied Models

To compare the five-fold cross-validation results of the SVR models, the evaluation indices of RSM based on the same data of the five-fold cross-validation approach were calculated and are shown in Table 6 and Table 7.

A comparison of the results reveals that the SVR model has a higher average R^2^ than the RSM model for both sensitivity and creep, indicating a better fit. The average RMSE and MAE of the SVR model for sensitivity and creep are lower than those of the RSM model, demonstrating that the SVR model has higher prediction accuracy.

**Figure 12:**
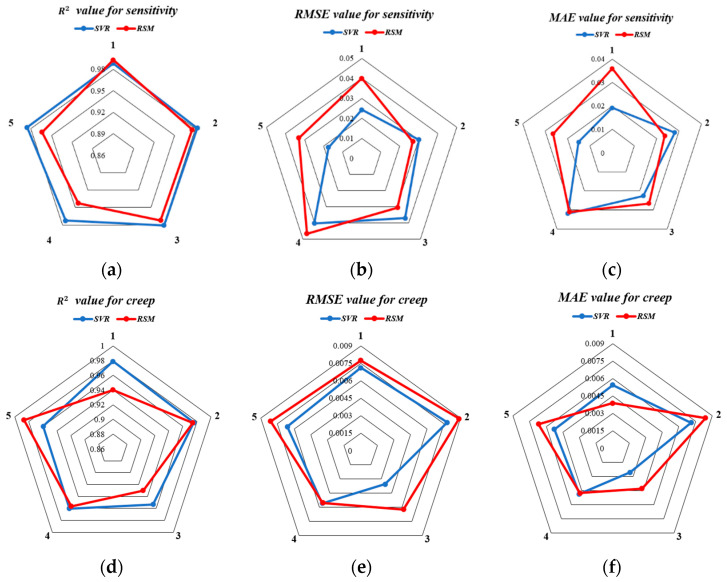
Radar diagrams of the five-fold cross-validation for the two estimation models in terms of $R^{2},\;RMSE,\;\mathrm{and}\;MAE$ for sensitivity and creep. (**a**) R^2^ value for sensitivity; (**b**) RMSE for sensitivity; (**c**) MAE for sensitivity; (**d**) R^2^ value for creep; (**e**) RMSE for creep; (**f**) MAE for creep.

Figure 12 shows the radar map of the five-fold cross-validation for the SVR and RSM model concerning R^2^, RMSE, and MAE. According to the following figures, the SVR model has higher R^2^ areas for both sensitivity and creep than the RSM model, and lower R^2^ areas for RMSE and MAE. This further suggests that the SVR model is more accurate in predicting sensitivity and creep. In light of this, the comparative analysis shows that both the RSM and SVR models exhibit a good fit and robustness toward performance prediction, with the SVR model having higher precision in comparison to the RSM model.

**Figure 13:**
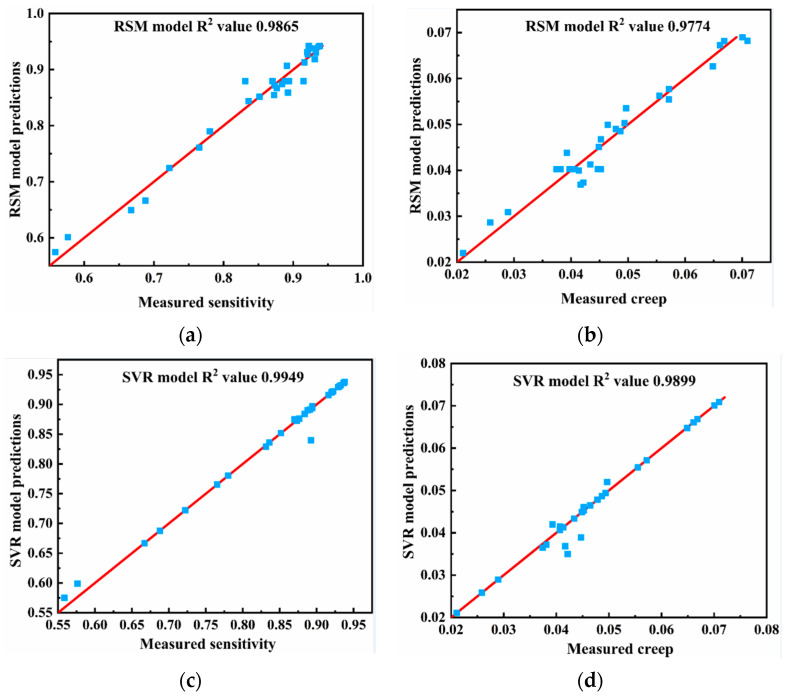
Comparison of the RSM and SVR performances for all data sets, where the red line represents the predicted value and the blue square represents the experimental value. (**a**) Sensitivity results for the RSM model; (**b**) creep results for the RSM model; (**c**) sensitivity results for the SVR model; (**d**) creep results for the SVR model.

Figure 13 depicts the linear dependency relationship between all experimental values and the anticipated value of the two models. According to Figure 13, the sensitivity R^2^ values for the RSM model and the SVR model are 0.9865 and 0.9949, respectively, and the creep R^2^ values are 0.9774 and 0.9899, respectively. As a result, both models’ predictions for the theoretical value and experimental value fit the data well; however, the SVR model’s predictions were closer to the measured values.

### 3.4. NSGA-II Results

**Figure 14:**
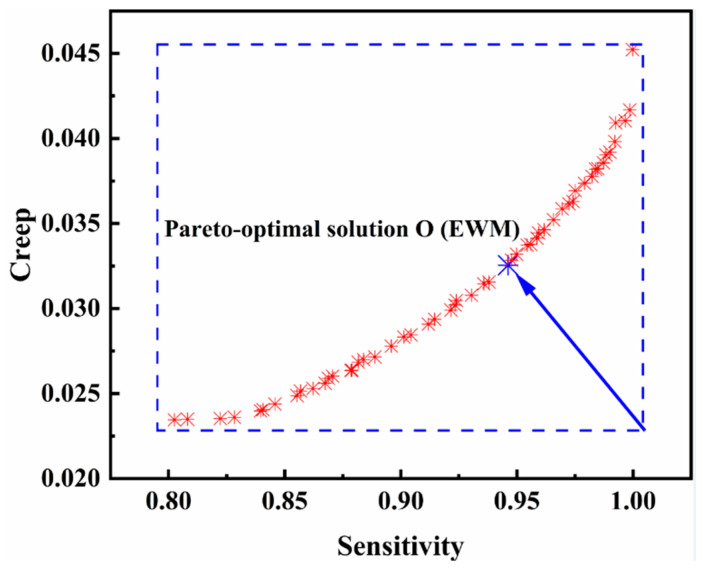
Pareto optimal solution.

The objective of the optimization method is to arrive at the material formula’s ideal ratio for sensitivity and creep. The SVR model was employed as a predictive model since its accuracy is superior to that of the RSM model, as was shown in Section 3.3. The NSGA -II algorithm eventually produced the Pareto optimum set. The non-convex multi-objective problem, which calls for appropriate parameters, can be solved with high accuracy and quick convergence using the NSGA-II method. The popular size in this study was 80, and the crossover ability and mutation probability were 0.05 and 0.8, respectively. The Pareto-optimal solutions are shown in Figure 14.

The sensitivity rises as creep increases, according to the Pareto front curve, and the final optimal solution was chosen using the entropy weight method (EWM), as illustrated in Figure 14. More specifically, the input parameters for the sensitivity, creep, and the magnetic field strength are as follows: Ni content = 2.392 g, MWCNT content = 0.767 g, MLG content = 1.468 g, and magnetic field intensity = 241 mT.

### 3.5. Validation Experiment and Comparison between RSM and SVR Model

All trials under the optimized conditions were carried out in quadruplicate, and the results are displayed in Table 8 to confirm the plausibility of the aforementioned analysis. Table 8 shows that both models can successfully fit the experimental data, with SVR performing better than RSM. The relative deviation is 0.317% for sensitivity and 0.307% for creep.

### 3.6. Model Fitting and Performance Testing

**Figure 15:**
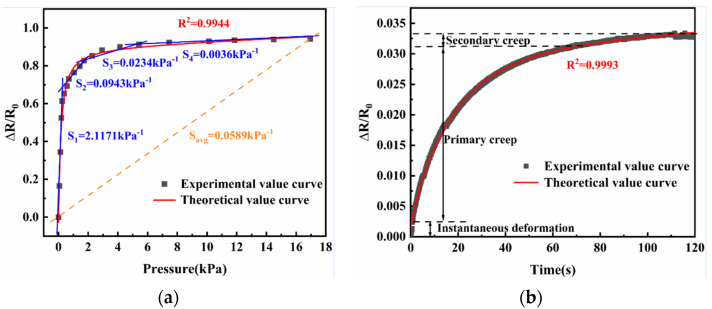
(**a**) Fitting curve and performance test for sensitivity and (**b**) fitting curve and performance test for creep.

As was previously indicated, the relative change in resistance, which is regarded as the sensitivity evaluation index, is the ‘sensitivity’ that was previously examined. The actual average sensitivity was 0.059 kPa^−1^, which is derived by dividing 0.9432 by 16 kPa. The actual sensitivity is the ratio of change in resistance and pressure. Figure 15 details the sensitivity and creep to demonstrate how the developed sensor performs. For sensitivity, at low pressure (<600 Pa), the sensitivity of the sensor was as high as 2.117 kPa^−1^. The sensor can cause significant deformation within this range. The sensor’s sensitivity in the medium pressure range (600–2000 Pa) was 0.09433 kPa^−1^. In the high pressure range (2000–6000 Pa), the sensitivity of the sensor was 0.0234 kPa^−1.^ After increasing the pressure, the deformation increment will decrease because of its rigidity. Thus, in the high pressure range (6000–16,000 Pa), the sensitivity of the sensor was 0.0036 kPa^−1^. When the pressure continued to increase, since the sensor has reached its saturation pressure, it cannot respond to external pressure efficiently. As a result, the sensor’s entire pressure range is 0–16 kPa, which increases the validity of the earlier assumption. As previously noted, the entire creep process can be observed to be divided into three parts: instantaneous deformation, primary creep, and secondary creep. As a result, the creep value is around 0.0324 when it progressively stabilizes after 120 s. Additionally, experimental data are fitted to the sensitivity and creep theoretical model examined in Section 2, and all of these fits exhibit high fitting accuracies.

Table 9 lists the sensitivity calculated in previous work. Compared with [26], the sensitivity of our sensor is in the same range, at 0.415 kPa^−1^ in the pressure range of 0–2 kPa. On comparison to reference [27], the sensitivity of our sensor is much larger, and reaches 0.141 kPa^−1^. In general, the comprehensive index of our sensor is greater than others because of its higher sensitivity and larger pressure range.

## 4. Conclusions

This study investigated the sensitivity and creep predictability of RSM and SVR methods and proposed the NSGA-II algorithm to improve fabrication conditions. The primary factors involved were magnetic field, Ni, MWCNTs, and MLGs. The results showed that all of the parameters had a significant impact on output performances. RSM dynamically detected the influence of numerous factors on performance. SVR was able to forecast sensitivity and creep using experimental data created by RSM as the input. When the results from SVR were compared to those from RSM, it was found that both methods had a high degree of output performance precision and that the predicted results from SVR were relatively close to the actual ones. This demonstrated the proposed method’s high ability to predict the complex relationships between multiple factors and output performances. All tests were run in quadruplicate under optimized conditions, and the optimal fabrication was determined using the NSGA-II algorithm. The suggested model’s excellent predictive capability was also demonstrated. Last but not least, the performance of our work was evaluated and compared with earlier work; the outcome reveals that our sensor performed better than others overall. This discovery offered a fresh perspective on artificial intelligence in transducer applications and has broad implications for calculating ideal conditions with minimal experiments, which can be seen as an enduring goal of researchers.

## Figures and Tables

**Table 1:** Previous work to improve sensor performance.

Reference	Filler Selection	Analyzed Parameter	Filler Type
[7]	CNT	Sensitivity	Single-particle
[8]	CNT	Creep	
[9]	MWCNT/Ni	Sensitivity	Two-particle
[10]	CNT/MLG	Sensitivity	
[11]	CNT/MLG	Creep	
[12]	MWCNT/Ni	Sensitivity	
[13]	CNT/Ni/PMXene	Sensitivity	Three-particle

**Table 2:** Levels and code of the four factors.

Code	Levels			
	Ni (g)	CNT (g)	MLG (g)	B (mT)
Low (−1)	1.6	0.6	0.8	210
Central (0)	2	0.8	1.2	280
High (1)	2.4	1	1.6	350

**Table 3:** Box–Behnken design (BBD) and experimental results.

Run	Factors				Sensitivity	Creep
	Ni	CNT	MLG	Magnetic Field Intensity		
1	−1	−1	0	0	0.6671	0.0211
2	−1	0	1	0	0.5765	0.0414
3	−1	0	0	−1	0.8516	0.0434
4	0	1	0	−1	0.9365	0.0555
5	0	0	1	1	0.6879	0.0572
6	−1	1	0	0	0.9310	0.0487
7	−1	0	−1	0	0.9290	0.0478
8	1	1	0	0	0.9380	0.0494
9	0	−1	1	0	0.5585	0.0258
10(C)	0	0	0	0	0.9147	0.0374
11	0	1	0	1	0.9159	0.0661
12(C)	0	0	0	0	0.8878	0.0452
13(C)	0	0	0	0	0.8940	0.0407
14	0	−1	0	−1	0.7802	0.0422
15	0	−1	−1	0	0.8764	0.0464
16	1	0	1	0	0.9326	0.0417
17	1	0	−1	0	0.8726	0.0649
18	1	−1	0	0	0.8732	0.0289
19	1	0	0	1	0.9210	0.0497
20	0	1	1	0	0.8910	0.0572
21	−1	0	0	1	0.7652	0.0452
22	1	0	0	−1	0.9202	0.0449
23(C)	0	0	0	0	0.8701	0.0381
24	0	0	−1	−1	0.8359	0.0668
25	0	−1	0	1	0.7222	0.0447
26	0	0	−1	1	0.9223	0.0709
27	0	1	−1	0	0.8839	0.0700
28(C)	0	0	0	0	0.8313	0.0398
29	0	0	1	−1	0.8923	0.0393

C indicates the central repeated trials.

**Table 4:** Value of the evaluation indices of the five-fold cross-validation for the SVR model on sensitivity.

Index	1	2	3	4	5	Average Value
R^2^	0.9882	0.9831	0.9805	0.9726	0.9857	0.9820
RMSE	0.02430	0.0301	0.0371	0.0403	0.0175	0.02985
MAE	0.01922	0.02807	0.02264	0.03205	0.01467	0.02339

**Table 5:** Value of the evaluation indices of the five-fold cross-validation for the SVR model on creep.

Index	1	2	3	4	5	Average Value
R^2^	0.9789	0.9757	0.9532	0.9504	0.9596	0.9656
RMSE	0.00711	0.00781	0.00356	0.00559	0.00662	0.006139
MAE	0.00544	0.00710	0.00258	0.00486	0.00526	0.005049

**Table 6:** Value of evaluation indices of the five-fold cross-validation for the RSM model on sensitivity.

Index	1	2	3	4	5	Average Value
R^2^	0.9929	0.9755	0.9721	0.9424	0.9640	0.9694
RMSE	0.03995	0.02694	0.03053	0.04671	0.03309	0.0354
MAE	0.03593	0.02363	0.02668	0.03106	0.02642	0.02874

**Table 7:** Value of evaluation indices of the five-fold cross-validation for the RSM model on creep.

Index	1	2	3	4	5	Average Value
R^2^	0.9401	0.9742	0.9301	0.9564	0.9870	0.9576
RMSE	0.00775	0.00888	0.00625	0.00555	0.00813	0.00731
MAE	0.00388	0.00840	0.00429	0.00478	0.00670	0.00561

**Table 8:** Verification of experiment results.

	Runs	Output	Mean ± SD	SVR Predicted Value	Relative Deviation (%)	RSM Predicted Value	Relative Deviation (%)
Sensitivity	1	0.9440	0.9432 ± 0.0011	0.9462	0.317	0.9510	0.82
	2	0.9432					
	3	0.9415					
	4	0.9444					
Creep	1	0.0328	0.0324 ± 0.000342	0.0325	0.307	0.0335	3.39
	2	0.0323					
	3	0.0327					
	4	0.0320					

**Table 9:** Comparison of sensitivity based on this work and previous reports.

Reference	Sensitivity (kPa^−1^)	Pressure Range (kPa)	Comparison (kPa^−1^)
[26]	0.438	0–2	0.415
[27]	0.02	0–6.5	0.141
[28]	0.23 × 10^−3^	0–3000	-
This work	0.0589	0–16	-

## Data Availability

The data presented in this study are available in this article.

## Supplementary Materials

The following supporting information can be downloaded at: https://www.mdpi.com/article/10.3390/nano13020298/s1. Table S1. The designed single-factor experiment; Table S2. ANOVA for the response of the sensitivity; Table S3. ANOVA for the response of the creep; Figure S1. The relationship between Ni content and performances. (a) Relationship between Ni content and creep; (b) relationship between Ni content and sensitivity; Figure S2. The relationship between MWCNT content and performances. (a) Relationship between MWCNT content and creep; (b) relationship between MWCNT content and sensitivity; Figure S3. The relationship between MLG content and performances. (a) Relationship between MLG content and creep; (b) relationship between MLG content and sensitivity; Figure S4. The relationship between magnetic field intensity and performances. (a) Relationship between magnetic field intensity and creep; (b) relationship between magnetic field intensity and sensitivity.

## Appendix A. Mathematical Formula

1. Calculations for Evaluating Modeling Accuracy

R^2^ indicates the coefficient of determination of a regression, RMSE represents the root mean squared error, and the MAE displays the mean absolute error, the specific equations are shown below:

(A1) $$R^{2}=1-\frac{\sum_{i=1}^{n}{\left(M_{i}-y_{i}\right)}^{2}}{\sum_{i=1}^{n}{\left(M_{i}-\bar{M}\right)}^{2}}$$

(A2) $$RMSE=\sqrt{\frac{1}{N}\overset{N}{\underset{i=1}{\sum }}{\left(M_{i}-y_{i}\right)}^{2}}$$

(A3) $$MAE=\frac{1}{N}\overset{N}{\underset{i=1}{\sum }}\left|M_{i}-y_{i}\right|$$

where $M_{i}$ and $y_{i}$ are the output performance of the $i_{th}$ fabrication condition measured via experiment and estimated via the proposed estimation models, respectively; $\bar{M}$ is the average output performance measured via experiment; and N is the total number of experimental conditions.

2. SVR model

The mathematical expression for SVR is shown below:

(A4) $$y=\overset{n}{\underset{i=1}{\sum }}w_{i}K\left(x,x_{i}\right)+b$$

where $w_{i}$ depicts the weight vector; $K\left(x,x_{i}\right)$ depicts the kernel function that solves quadratic equations linearly; and b represents the bias.

By introducing slack variables (*ξ* and $\xi^{*}$) with a scalar constant C, the optimization problem is obtained:

(A5) $$Minimize\;\frac{\|w{\|}^{2}}{2}+C\overset{N}{\underset{i=1}{\sum }}\left(\xi_{i}+\xi_{i}^{*}\right)$$

(A6) $$Subjected\;to\left\{\begin{array}{c} y_{i}-\langle w\cdot K\left(x,x_{i}\right)\rangle -b\leq \varepsilon +\xi_{i}\\ \langle w\cdot K\left(x,x_{i}\right)\rangle +b-y_{i}\leq \varepsilon +\xi_{i}^{*}\\ \xi_{i},\xi_{i}^{*}\geq 0 \end{array}\right.$$

By applying Lagrange multipliers ($\alpha_{i}$ and $\alpha_{i}^{*}$), the dual space transformation is shown in Equations (A7) and (A8):

(A7) $$Maximize\frac{1}{2}\overset{N}{\underset{i,j=1}{\sum }}\left(\alpha_{i}-\alpha_{i}^{*}\right)\left(\alpha_{j}-\alpha_{j}^{*}\right)K\left(x_{i},x_{j}\right)-\varepsilon \overset{N}{\underset{i=1}{\sum }}\left(\alpha_{i}-\alpha_{i}^{*}\right)+\overset{N}{\underset{i=1}{\sum }}E_{i}\left(\alpha_{i}-\alpha_{i}^{*}\right)$$

(A8) $$Subjected\;to\left\{\begin{array}{c} \sum_{i=1}^{N}\left(\alpha_{i}-\alpha_{i}^{*}\right)=0\\ 0\leq \alpha_{i}\leq C\\ 0\leq \alpha_{i}^{*}\leq C \end{array}\right.$$

Based on the above analysis, the final SVR model can be obtained:

(A9) $$y=\overset{n}{\underset{i=1}{\sum }}\left(\alpha_{i}-\alpha_{i}^{*}\right)K\left(x,x_{i}\right)+b$$
