# Supplementary material for: Modeling and Optimization of Sensitivity and Creep for Multi-Component Sensing Materials
Source: Nanomaterials (Basel). 2023 Jan 11;13(2):298. doi: 10.3390/nano13020298 (PMC9862774; doi:10.3390/nano13020298)
Supplement: Supplementary file 1 [file nanomaterials-13-00298-s001.zip › nanomaterials-2058478-supplementary.pdf]

Supplementary Materials

1. Single factor experiment

The single-factor experiment was designed by the control variable method. The factor variables were contents of Ni (0-4g), MWCNTs (0-1g) and MLGs (0-2.4g), and magnetic field intensity (0-420mT) based on previous experimental preparation. When increasing one content of a single factor, the other contents were at average values, the specifically designed scheme was shown in Table S1.

Table S1. The designed single-factor experiment.

No.	Ni(g)	MWCNTs(g)	MLGs(g)	B(mT)
1	0,0.8,1.6,2,2.4,3.2,4	0.5	1.2	210
2	2	0,0.2,0.4,0.5,0.6,0.8,1	1.2	210
3	2	0.5	0,0.4,0.8,1.2,1.6,2,2.4	210
4	2	0.5	1.2	0,70,140,210,280,350,420

1.1 Single factor analysis of Ni

Ni content is one of the main factors affecting the performance of the sensor. throughout this experiment, MWCNT and MLG content remains constant. It can be seen from Figure S1 that in terms of creep, the creep gradually increases with the increase of Ni content and reaches the largest value when the content is 7.13%. In terms of sensitivity, the sensitivity gradually increases with the increase of Ni content and reaches the highest value when content is 7.13%. Since the increase in Ni content will reduce the creep resistance and increase the sensitivity, the Ni content range is selected in the middle range, that is, 3.7%-5.44% to ensure that the final sample has both high sensitivity and strong creep resistance.

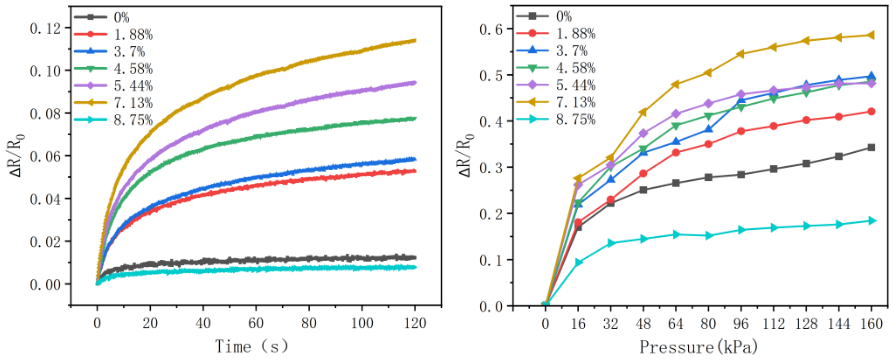

(a) (b)

Figure S1. The relationship between Ni content and performances. (a) Relationship between Ni content and creep; (b) relationship between Ni content and sensitivity.

1.2 Single factor analysis of MWCNT

Similar to the previous analysis, other variables are fixed when analyzing MWCNTs. From Figure S2, in terms of creep, the creep is the least when the CNT content is in the range of 1.14%-1.37%. In terms of sensitivity, when the CNT content exceeds 1.37%, the sensitivity rises sharply to a higher level. Therefore, to keep high sensitivity and low creep, the best content for creep is selected as the lower limit and the content for the highest sensitivity is considered as the upper limit. Thus, the range of CNTs is selected between

1.37% and 2.26% to ensure good performance. At the same time, as shown in Figure S2(b), when the content of CNTs is high, their sensitivity gradually decreases with the increase of pressure, which will limit the measurement range of the sensor, but this situation can be applied to the field of early warning.

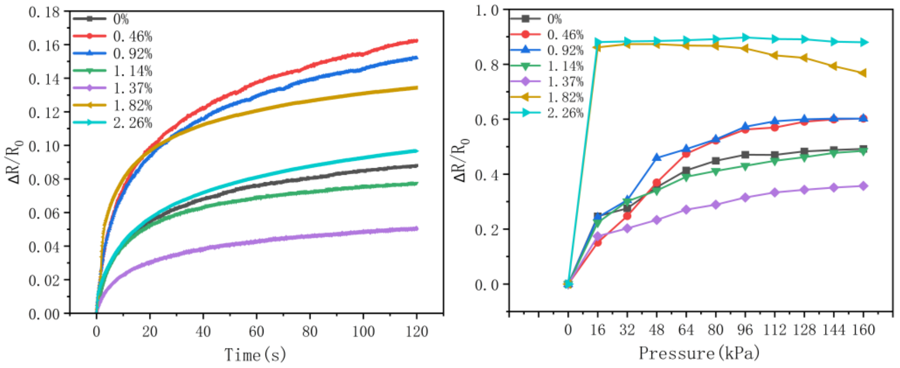

(a) (b)

Figure S2. The relationship between MWCNT content and performances. (a) Relationship between MWCNT content and creep; (b) relationship between MWCNT content and sensitivity.

1.3 Single factor analysis of MLG

As shown in Figure S3, in terms of creep, the creep decreases with the increase of MLG content. In terms of sensitivity, when the MLG content is within 3.36%, the sensitivity gradually increases with the increase of graphene content. Gradually increasing the pressure, its sensitivity also changes, and the sensitivity is the greatest when the graphene content is 1.85%. When the graphene content exceeds 3.36%, although the sensitivity is improved under low pressure, the overall measurement range is narrowed. Therefore, the content range of graphene is 1.85%-3.36% as the reference range for subsequent experiments.

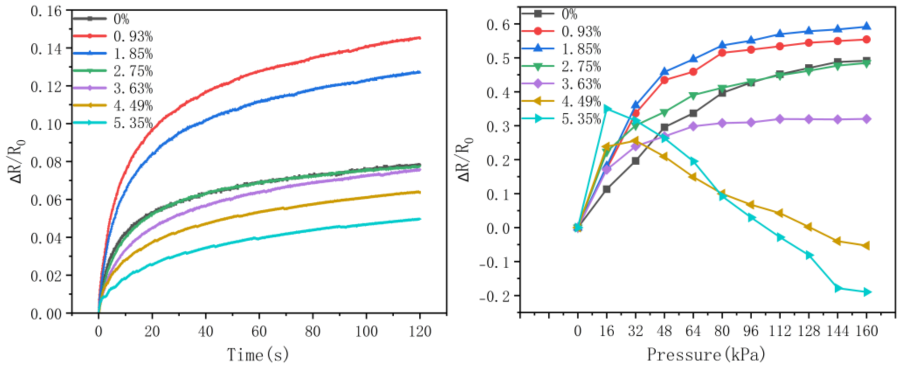

(a) (b)

Figure S3. The relationship between MLG content and performances. (a) Relationship between MLG content and creep; (b) relationship between MLG content and sensitivity.

1.4 Single-factor analysis of magnetic field intensity

As shown in Figure S4, it can be observed that the magnetic field intensity has a significant influence on creep and sensitivity. In terms of creep, when the magnetic field intensity is small, its creep is gradually increased with the enhancement of the magnetic field. When the magnetic field intensity is between 210mT-350mT, the creep reaches the lowest value when the intensity is 280mT. In terms of sensitivity, the sensitivity achieves a maximum value when the intensity is between 210mT-380mT. Therefore, based on the

creep and sensitivity results, the magnetic field range of 210mT-380mT was selected as the experimental range of subsequent experiments.

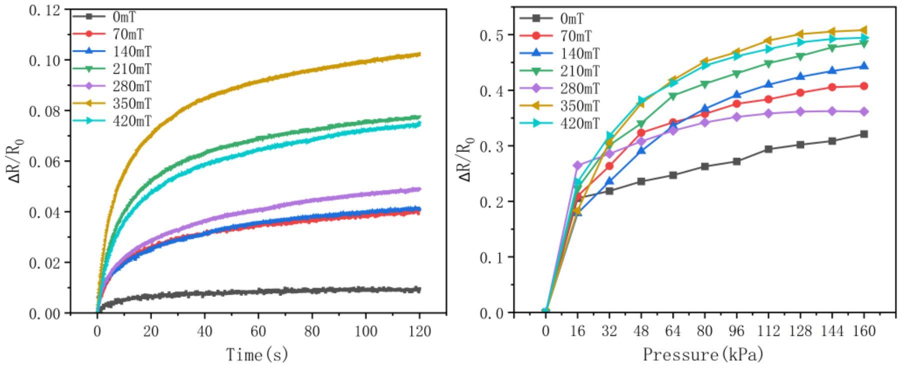

(a) (b)

Figure S4. The relationship between magnetic field intensity and performances. (a) Relationship between magnetic field intensity and creep; (b) relationship between magnetic field intensity and sensitivity.

To sum up, from the above analysis, the resistance creeps become stable within the 120s. The change in resistance increases before 16 kPa for all situations. Thus, the measured range for creep is 120s and the range of sensitivity is assumed as 0-16kPa for the following analysis.

2. ANOVA for the response of sensitivity and creep.

This single factor has a significant influence on sensitivity. In the interaction term, the interaction between the Ni and MLG contents, the interaction between MWCNT and MLG contents, the interaction between Ni and MWCNT contents and the interaction between MLG and B had extremely significant influences on sensitivity; the interaction between Ni and MWCNT contents had significant influences on sensitivity. In the square items, the square term of CNT and MLG contents had significant influences on the sensitivity.

Table S2. ANOVA for the response of the sensitivity.

Source	Sum of squares	Degree of Freedom	Mean squares	F-value	P-value	Status	Summary of the Statistics
Model	0.3077	14	0.022	36.4	<0.0001	ss	
x_1-Ni	0.0453	1	0.0453	74.99	<0.0001	ss	
x_2-CNT	0.0865	1	0.0865	143.22	<0.0001	ss	
x_3-MLG	0.0509	1	0.0509	84.27	<0.0001	ss	
x_4-B	0.0066	1	0.0066	11.00	0.0051	s	
x_1x_2	0.0099	1	0.0099	16.38	0.0012	s	
x_1x_3	0.0426	1	0.0426	70.47	<0.0001	ss	
x_1x_4	0.0019	1	0.0019	3.14	0.0980	ns	$R^2 - 0.9865$
x_2x_3	0.0264	1	0.0264	43.73	<0.0001	ss	$Adj\_R^2 - 0.947$
x_2x_4	0.0003	1	0.0003	0.5791	0.4593	ns	$C.V.\% - 2.91$
x_3x_4	0.0211	1	0.0211	35.01	<0.0001	ss	
x_1^2	0.0001	1	0.0001	0.1920	0.6679	ns	
x_2^2	0.0057	1	0.0057	9.50	0.0081	s	
x_3^2	0.0126	1	0.0126	20.91	0.0004	ss	
x_4^2	0.0004	1	0.0004	0.6157	0.4457	ns	
Lack of Fit	0.0045	10	0.0005	0.4605	0.8541	ns	
Pure Error	0.0039	4	0.0010				

In the linear term, all the input parameters had an extremely significant effect on creep. In the interaction term, only the interaction between the Ni and MLG contents had significant influences on creep, while the remaining interaction items had no significant influences. In the square items, the square term of B, and MLG contents had extremely significant influences on the creep, while the other items had no significant influences.

Table S3. ANOVA for the response of the creep.

Source	Sum of squares	Degree of Freedom	Mean squares	F-value	P-value	Status	Summary of the Statistics
Model	4.2×10^{-3}	14	3×10^{-4}	21.34	<0.0001	ss	
x_1-Ni	1×10^{-4}	1	1×10^{-4}	6.06	0.0274	s	
x_2-CNT	1.6×10^{-3}	1	1.6×10^{-3}	113.46	<0.0001	ss	
x_3-MLG	9×10^{-4}	1	9×10^{-4}	65.28	<0.0001	ss	
x_4-B	1×10^{-4}	1	1×10^{-4}	10.45	0.0060	ss	
x_1x_2	1.29×10^{-5}	1	1.29×10^{-5}	0.9247	0.3525	ns	
x_1x_3	1×10^{-4}	1	1×10^{-4}	5.00	0.0421	s	
x_1x_4	2.18×10^{-6}	1	2.18×10^{-6}	0.1567	0.6982	ns	$R^2 - 0.9774$
x_2x_3	1.49×10^{-5}	1	1.49×10^{-5}	1.07	0.3183	ns	$Adj\_R^2 - 0.911$
x_2x_4	1.64×10^{-5}	1	1.64×10^{-5}	1.17	0.2969	ns	
x_3x_4	4.78×10^{-5}	1	4.78×10^{-5}	3.43	0.0851	ns	$C.V.\% - 7.898$
x_1^2	5.73×10^{-5}	1	1×10^{-4}	4.11	0.0620	ns	
x_2^2	2.86×10^{-6}	1	2.86×10^{-6}	0.2052	0.6575	ns	
x_3^2	6×10^{-4}	1	6×10^{-4}	45.16	<0.0001	ss	
x_4^2	6×10^{-4}	1	6×10^{-4}	41.06	<0.0001	ss	
Lack of Fit	2×10^{-4}	10	1.57×10^{-5}	1.67	0.3278		
Pure Error	3.77×10^{-5}	4	9.42×10^{-6}				
